# Pan-Cancer Interrogation of B7-H3 (*CD276*) as an Actionable Therapeutic Target Across Human Malignancies

**DOI:** 10.1158/2767-9764.CRC-23-0546

**Published:** 2024-05-30

**Authors:** Carly D. Miller, John R. Lozada, Nicholas A. Zorko, Andrew Elliott, Allison Makovec, Milan Radovich, Elisabeth I. Heath, Neeraj Agarwal, Rana R. Mckay, Rohan Garje, Bruno R. Bastos, Dave S.B. Hoon, Jacob J. Orme, Oliver Sartor, Ari VanderWalde, Chadi Nabhan, George Sledge, Eugene Shenderov, Scott M. Dehm, Emil Lou, Jeffrey S. Miller, Justin H. Hwang, Emmanuel S. Antonarakis

**Affiliations:** 1 Masonic Cancer Center, University of Minnesota, Minneapolis, Minnesota.; 2 Caris Life Sciences, Phoenix, Arizona.; 3 Karmanos Cancer Institute, Detroit, Michigan.; 4 Huntsman Cancer Institute, University of Utah, Salt Lake City, Utah.; 5 University of California San Diego, La Jolla, California.; 6 Miami Cancer Institute, Baptist Health South Florida, Miami, Florida.; 7 Saint John's Cancer Institute PHS, Santa Monica, California.; 8 Mayo Clinic Comprehensive Cancer Center, Rochester, Minnesota.; 9 The Sidney Kimmel Comprehensive Cancer Center, Johns Hopkins School of Medicine, Baltimore, Maryland.; 10 Departments of Laboratory Medicine and Pathology and Urology, University of Minnesota, Minneapolis, Minnesota.

## Abstract

B7-H3 (*CD276*) is a transmembrane glycoprotein of the B7 immune checkpoint superfamily that has emerged as a promising therapeutic target. To better understand the applicability of B7-H3–directed therapies, we analyzed 156,791 samples comprising 50 cancer types to interrogate the clinical, genomic, transcriptomic, and immunologic correlates of B7-H3 mRNA expression. DNA (592-gene/whole-exome) and RNA (whole-transcriptome) sequencing was performed from samples submitted to Caris Life Sciences. B7-H3 high versus low expression was based on top and bottom quartiles for each cancer type. Patients’ overall survival was determined from insurance claims data. Pathway analysis was performed using gene set enrichment analyses. Immune cell fractions were inferred using quanTIseq. B7-H3 is expressed across several human malignancies including prostate, pancreatic, ovarian, and lung cancers. High B7-H3 expression is associated with differences in overall survival, possibly indicating a prognostic role of B7-H3 for some cancers. When examining molecular features across all cancer types, we did not identify recurrent associations between B7-H3 expression and genetic alterations in *TP53*, *RB1*, and *KRAS*. However, we find consistent enrichment of epithelial-to-mesenchymal transition, Wnt, TGFβ, and Notch signaling pathways. In addition, tumors with high B7-H3 expression are associated with greater proportions of M1 macrophages, but lower fractions of CD8^+^ T cells. We have begun to define the genomic, transcriptomic, clinical, and immunologic features associated with B7-H3 expression in 50 cancer types. We report novel clinical and molecular features of B7-H3–high tumors which may inform how current B7-H3 therapeutics should be deployed and prioritized.

**Significance:**

B7-H3–targeting therapeutics have shown promising results in initial clinical trials. In this pan-cancer analysis of B7-H3 mRNA expression, we found that B7-H3 exhibits robust expression in many common cancer types. These results may inform further development of B7-H3–targeting therapeutics and may guide clinical decisions for patients with limited treatment options.

## Introduction

In recent years, targeted cancer therapies have gained popularity due to their ability to target both solid tumors and hematologic malignancies refractory to traditional therapies (1). Immune checkpoint inhibitors (ICI) targeting PD-1, PD-L1, and CTLA-4 have become widely utilized because of their efficacy in various clinical contexts where traditional treatments have failed (2). ICIs have shown success in the treatment of head and neck cancers, non–small cell lung cancer (NSCLC), melanoma, renal cell carcinoma, and others (3). Nonetheless, current ICIs still do not exhibit efficacy in all tumors, with intratumoral heterogeneity and immunosuppressive tumor microenvironments acting as barriers to effective responses (4). Furthermore, in the vast majority of ICI treated tumors, treatment is noncurative. Such limitations warrant the discovery of new immunotherapy targets and strategies to enhance response in tumors where current ICIs have a suboptimal response.

Recently, B7-H3 (*CD276*) has emerged as a promising target for a variety of cancers. B7-H3 is a transmembrane glycoprotein within the B7 immune checkpoint superfamily, which includes PD-L1 and CTLA-4 (5). B7-H3 is expressed on both immune cells and many solid tumors and has been found to have both costimulatory and coinhibitory roles in the regulation of T cells (6, 7). Many studies have described overexpression of B7-H3 in various cancers, including leukemias, prostate cancer, ovarian cancer, pancreatic cancer, colorectal cancers, melanoma, and others (8–10). This overexpression directly on tumor cells (TC) is thought to drive tumor progression and correlate with poor prognosis (11, 12). Of note, targeting B7-H3 using antibody–drug conjugates (ADC) has been shown to have potential efficacy in treating prostate cancer (13, 14).

With emerging evidence of B7-H3 expression in the pan-cancer setting, there have been numerous advances in the development of B7-H3 targeted therapies. For example, enoblituzumab, a B7-H3–targeting Ab, has shown potential in early clinical trials (NCT02923180) as a neoadjuvant immunotherapeutic option for patients with high-grade prostate cancer (15). Given that B7-H3 is associated with metastasis and recurrence in prostate cancer, these targeted therapies are also being deployed in metastatic prostate cancer clinical trials. Aside from mAbs, B7-H3–directed ADCs and immune engagers have shown efficacy in pediatric solid tumors and NSCLC, respectively (16, 17). In addition, past work has reported that B7-H3 may serve as a glioblastoma biomarker and potential target (18). Consequently, chimeric antigen receptor T-cell therapies targeting B7-H3 have been developed against pediatric solid tumors and there is currently enrollment of patients with recurrent glioblastoma multiforme in a phase I trial (NCT05474378; ref. 19). Considering the increasing interest in B7-H3 as a therapeutic target, it is critical to understand its functions and applicability in a broad range of tumor types.

Here, we conducted a comprehensive interrogation of B7-H3 expression in a pan-cancer dataset aggregated by the Caris Precision Oncology Alliance consortium. Our work aimed to investigate the genomic, transcriptomic, clinical, and immunologic features associated with B7-H3 expression in more than 50 cancer types in over 156,000 tumor samples. Novel clinical and molecular features we have uncovered in these tumors inform how the use of current B7-H3 therapeutics may be expanded in multiple human malignancies.

## Materials and Methods

### Specimens

We queried the Caris Life Sciences database to assess molecular alterations and related survival outcomes of 156,791 tumor biopsies across 50 human cancer types. Comprehensive molecular profiling was performed in a CLIA/CAP/ISO15189 certified clinical laboratory (Caris Life Sciences; ref. 20). This study was conducted in accordance with the guidelines of the Declaration of Helsinki, Belmont Report, and U.S. Common Rule. In keeping with 45 CFR 46.101(b)(4), this study was carried out using retrospective deidentified clinical data. Therefore, patient consent was waived, and this study was considered exempt at each Institutional Review Board.

### Survival Analysis

We queried the deidentified real-world evidence (RWE) outcomes dataset from the Caris Life Sciences Precision Oncology Alliance registry and insurance claims data. RWE overall survival (OS) was defined as date of treatment initiation to either the date of death or last contact in the insurance claims repository. As reported previously, patient death was assumed for any patient without a claim for more than 100 days, which holds true for more than 95% of patients with a recorded death in the National Death Index. Cox proportional hazard ratios (HRs) were calculated for each comparison group and significance was determined as *P* values of < 0.05 using the log-rank statistic.

### DNA Next-generation Sequencing

Direct sequence analysis was conducted on genomic DNA isolated from microdissected, formalin-fixed, paraffin-embedded (FFPE) tumor samples using the Illumina Nextseq (592-gene panel) or Novaseq 6000 (whole-exome) sequencers. A hybrid pulldown of baits designed to enrich for 592 clinically relevant genes at high coverage and high read-depth was used, along with another panel designed to enrich for an additional >20k genes at a lower depth for samples profiled by whole-exome sequencing, which included a 500 Mb SNP backbone panel (Agilent technologies) was added to help with gene amplification/deletion detection as described previously (20).

### Whole-transcriptome Sequencing

Qiagen RNA FFPE tissue extraction kit was used for extraction. RNA quality and quantity were determined using the Agilent TapeStation (Agilent TapeStation Laptop, RRID:SCR_019547). Biotinylated RNA baits were hybridized to the synthesized and purified cDNA targets, and the bait–target complexes were amplified in a postcapture PCR reaction. The Illumina NovaSeq 6500 was used to sequence the whole transcriptome from patients to an average of 60M reads. Raw data were demultiplexed by the Illumina Dragen BioIT accelerator, trimmed, counted, removed of PCR duplicates, and aligned to human reference genome hg19 by the STAR aligner. For transcription counting, transcripts per million molecules were generated using the Salmon expression (20).

### Protein IHC

An automated staining platform (Ventana Medical Systems, Inc.) was used to detect PD-1/PD-L1 protein expression by IHC: the SP142 Ab (laboratory developed test) was used to stain for PD-L1 expression on TCs of prostate, colorectal, ovarian, and pancreatic cancers, with a positive threshold of ≥2+ intensity and ≥5% cell stained; the SP142 Ab (Ventana) was used to stain for PD-L1 on immune cells of breast cancer, with a positive threshold of ≥1% of cells stained; the 22c3 Ab was used in NSCLC and ovarian cancers, with a positive threshold of tumor proportion score (TPS) ≥1 and combined positive score (CPS) ≥1, respectively; the 28-8 Ab was used to stain for PD-L1 expression on TCs for NSCLC, with a positive threshold of ≥1+ intensity and ≥1% cell stained; and the PD-1 Ab was used for ovarian cancer, with a positive threshold of ≥1% cells stained. Benign tonsil samples served as a positive control. MMR protein expression was tested by IHC (Ventana Medical Systems) using Ab clones for the four mismatch repair proteins [MLH1, M1 Ab; MSH2, G2191129 Ab; MSH6, 44 Ab; and PMS2 (Abcam, catalog no. ab203457, RRID:AB 2889230), EPR3947 Ab (Ventana Medical Systems, Inc.)]. The complete absence of protein expression of any of the four proteins tested (0+ in 100% of cells) was considered deficient MMR as described previously (21).

### Microsatellite Instability

Microsatellite instability-high (MSI-H) was determined by next-generation sequencing, with >2,800 target microsatellite loci examined and compared with the reference genome hg19 from the University of California, Santa Cruz (UCSC) Genome Browser database.

### Tumor Mutation Burden

Tumor mutation burden (TMB) was measured by counting all nonsynonymous missense, nonsense, in-frame insertion/deletion, and frameshift mutations found per tumor that had not been previously described as germline alterations in dbSNP151, Genome Aggregation Database (gnomAD) databases, or benign variants identified by Caris geneticists. A cut-off point of ≥10 mutations per megabase (mt/Mb) was used on the basis of the KEYNOTE-158 pembrolizumab trial (22).

### Immune Cell Deconvolution

To examine the potential associations of B7-H3 and unique tumor immune landscapes, CD8^+^ T cells, M1 macrophage, and M2 macrophage tumor infiltration was inferred using quanTIseq, a computational method for estimating immune cell fractions by deconvolution of bulk RNA sequencing (RNA-seq) data (23).

### Statistical Analyses

Statistical analyses were performed using JMP version 16.1.0 (SAS Institute Inc.) and standard Python packages (NumPy and SciPy), along with other packages for plotting (Pandas, Seaborn, and Matplotlib). Statistical significance was determined using X2 and Mann–Whitney *U* tests with corrections for multiple comparisons (Benjamini–Hochberg) where appropriate.

### Data Availability Statement

The datasets generated and/or analyzed during the current study are available from the corresponding author on reasonable request. The deidentified DNA/RNA-seq data are protected proprietary information owned by Caris Life Sciences, and qualified researchers can apply for access to these summarized data by signing a data usage agreement. However, these data cannot be deposited into public repositories. Inquiries can be sent to Andrew Elliott at aelliott@carisls.com

## Results

### The Spectrum of B7-H3 Expression Across Human Malignancies

**FIGURE 1:**
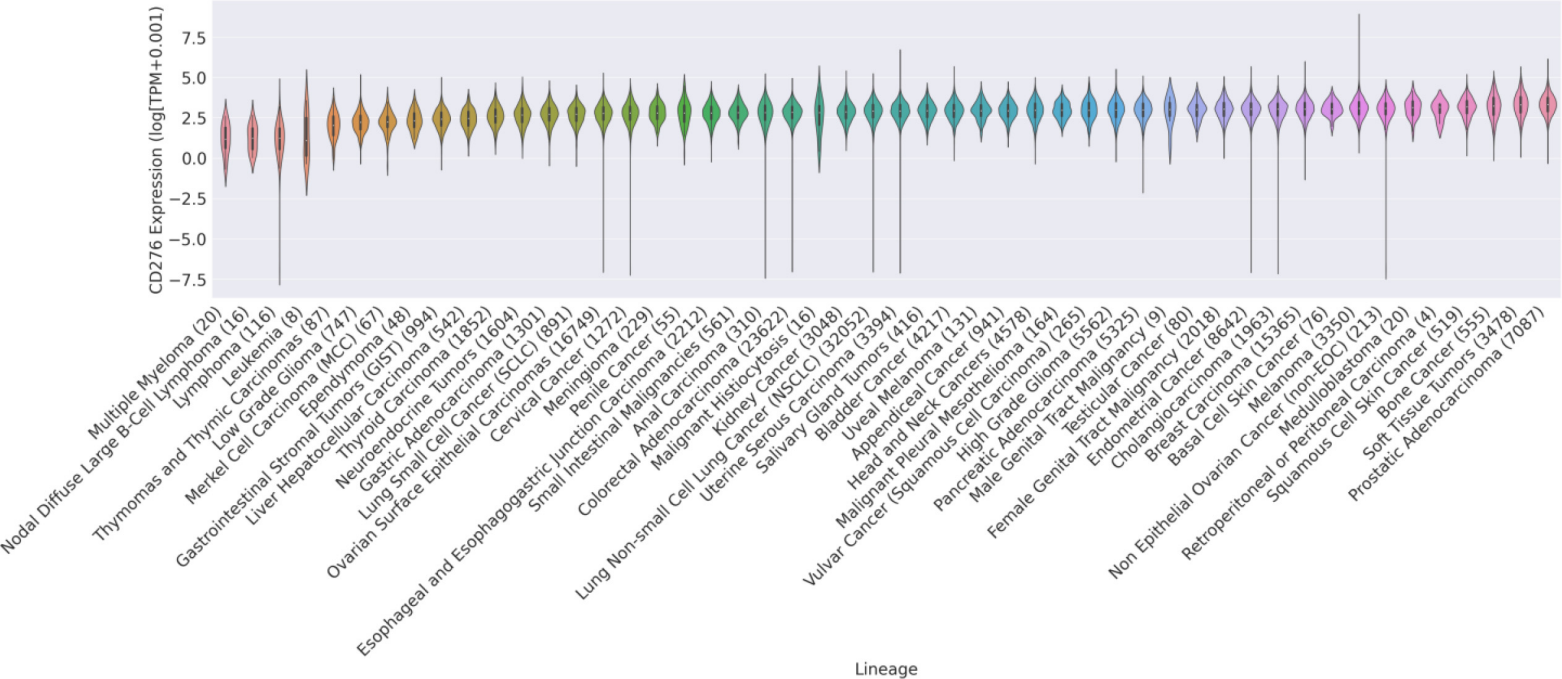
Characterization of B7-H3 expression in tumors. B7-H3 transcripts per million (TPM) after log normalization. A total of 156,791 samples from 50 distinct tumor types were analyzed from the Precision Oncology Alliance (POA) consortium. Data shown in violin plots in which the white dot represents the median and the black box shows the ends of the first and third quartiles. The female genital tract malignancy category represents sarcomas from gynecologic sites and the male genital tract malignancy category represents primarily testicular cancer.

Our group has previously demonstrated B7-H3 (*CD276*) to be significantly elevated in metastatic prostate cancers compared with primary prostate cancer (8). In studying the levels of B7-H3 expression in the transcriptomes of 369 cancer cell lines spanning 35 cancer types in the Cancer Cell Line Encyclopedia, we further showed that mRNA levels have a strong positive correlation with protein levels in cancer cell lines, as well as in patient-derived xenograft models derived from patients with prostate cancer (24). This association indicates that *CD276* transcript levels are a reasonable surrogate for B7-H3 protein expression. Therefore, we then examined the transcriptomes of 156,791 tumors spanning 50 cancer types, detecting robust expression of B7-H3 in many cancers, including sarcomas, prostate, ovarian, breast, and pancreatic cancers (Fig. 1). Notably, sarcomas and prostate tumors displayed the highest median expression levels [26.9 transcripts per million (TPM) and 26.0 TPM, respectively], with 52.5% of sarcomas and 48.8% of prostate cancers being categorized as B7-H3 high (top 25% globally pan-cancer). In contrast, hematologic malignancies, such as lymphomas and multiple myeloma, displayed the lowest expression of B7-H3 across all cancer types. Across cancer types, we also looked at differences between B7-H3 expression and age (greater than vs. less than 65 years old; Supplementary Fig. S1A) as well as sex (male vs. female; Supplementary Fig. S1B).

### B7-H3 Expression is Differentially Associated with Clinical Outcomes

**FIGURE 2:**
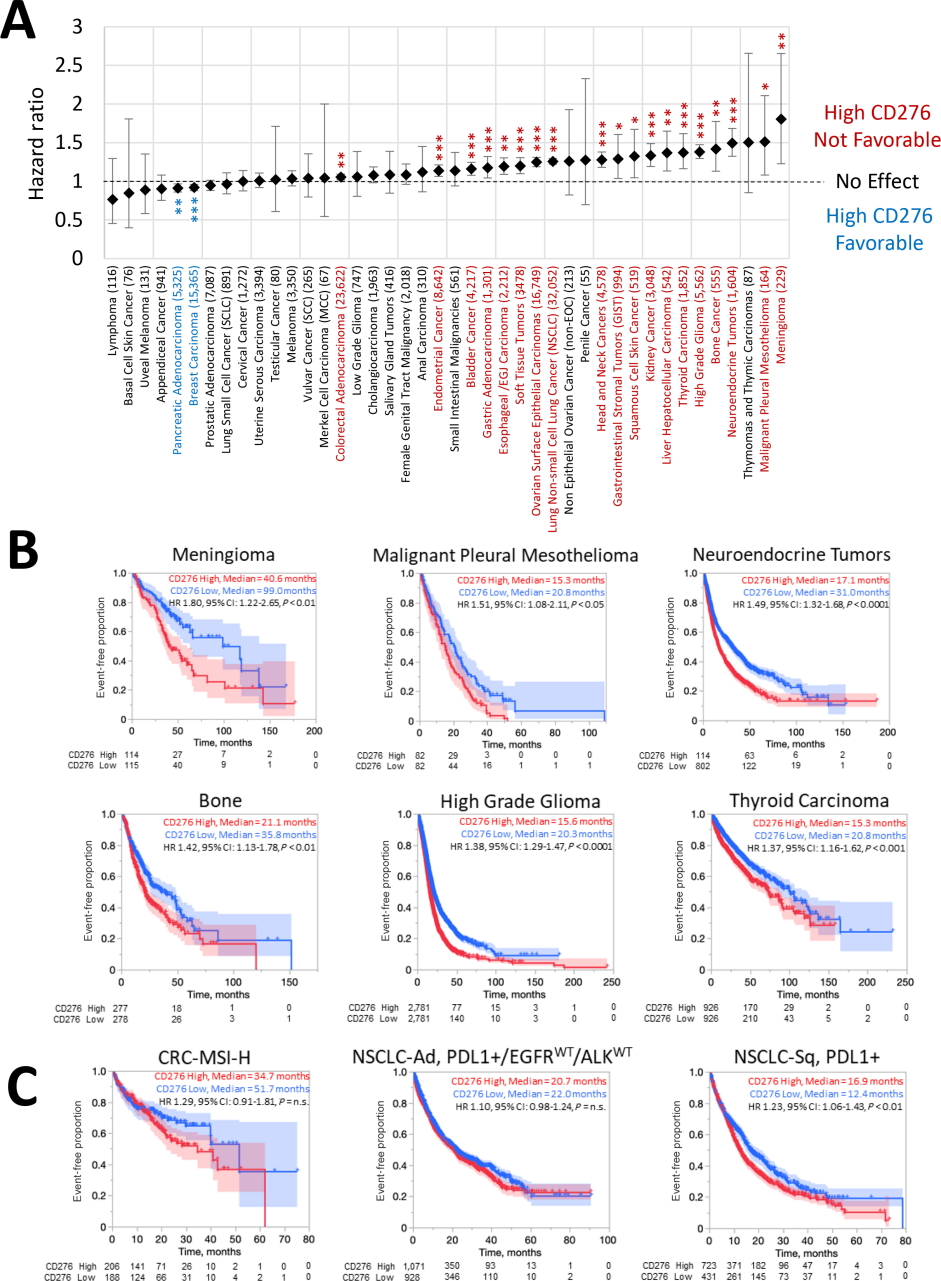
Tumor profile association with OS. **A,** Association of OS in tumors with the top 75th percentile of B7-H3 expression (relative to all cancers) across 50 tumor types. *, *q* < 0.05; **, *q* < 0.01; ***, *q* < 0.001. **B,** OS of patients with high B7-H3 expression compared with low B7-H3 expression (stratified on the basis of medians) for the top six cancers where CD276-high expression is associated with poor OS. Cox proportional HRs were calculated for each comparison group with significance determined as *P* values of <0.05 using log-rank statistics. **C,** OS of patients with high B7-H3 expression compared with low B7-H3 expression (stratification based on quartiles) from the start of pembrolizumab treatment for patients with colorectal cancer with microsatellite instability (CRC-MSI-H), NSCLC-adenocarcinoma (NSCLC-Ad) that is PD-L1–positive/EGFR wildtype/ALK wildtype, and NSCLC-squamous (NSCLC-Sq) cell carcinoma that is PD-L1 positive.

Previous studies have demonstrated that B7-H3 expression may serve as a prognostic marker associated with worse OS, albeit controversial in several cancer types, including pancreatic, prostatic, and colorectal cancers (25–28). Given our large sample sizes in most cancers, in conjunction with the uniform processing and handling of all samples, we sought to investigate the association between B7-H3 expression and OS in our cohorts (Fig. 2A). We analyzed each cancer type separately and determined B7-H3–high versus –low cutoffs based on the median expression value within the given cancer type. These thresholds were also applied to further downstream analyses. For several cancer types, we found that high B7-H3 expression predicted worse OS. These included colorectal adenocarcinoma, endometrial cancer, bladder cancer, gastric adenocarcinoma, esophageal carcinoma, soft-tissue tumors, ovarian surface epithelial cancers, NSCLCs, head and neck cancers, gastrointestinal stromal tumors, squamous cell skin cancer, kidney cancer, hepatocellular cancer, thyroid carcinoma, high-grade gliomas, bone cancers, neuroendocrine tumors, malignant pleural mesotheliomas, and meningiomas. In contrast, we made the unexpected finding that high B7-H3 expression predicted better OS in pancreatic adenocarcinoma and breast cancers (Fig. 2B). We also looked at OS from the start of treatment with pembrolizumab for several cancer types. We considered colorectal cancer with microsatellite instability (MSI), lung adenocarcinoma that is PD-L1 positive, EGFR wildtype, and ALK wildtype, as well as lung squamous cell carcinoma that is PD-L1 positive (Fig. 2C). For lung squamous cell carcinoma with PD-L1 positivity, there was greater OS for B7-H3–high cancers compared with B7-H3–low tumors after initiating pembrolizumab (HR, 1.23; 95% confidence interval: 1.06–1.43; *P* < 0.01). There were nonsignificant differences in survival of colorectal cancer and lung adenocarcinoma after pembrolizumab treatment.

### Classical Immunotherapy Biomarkers are not Associated with B7-H3 Status

**FIGURE 3:**
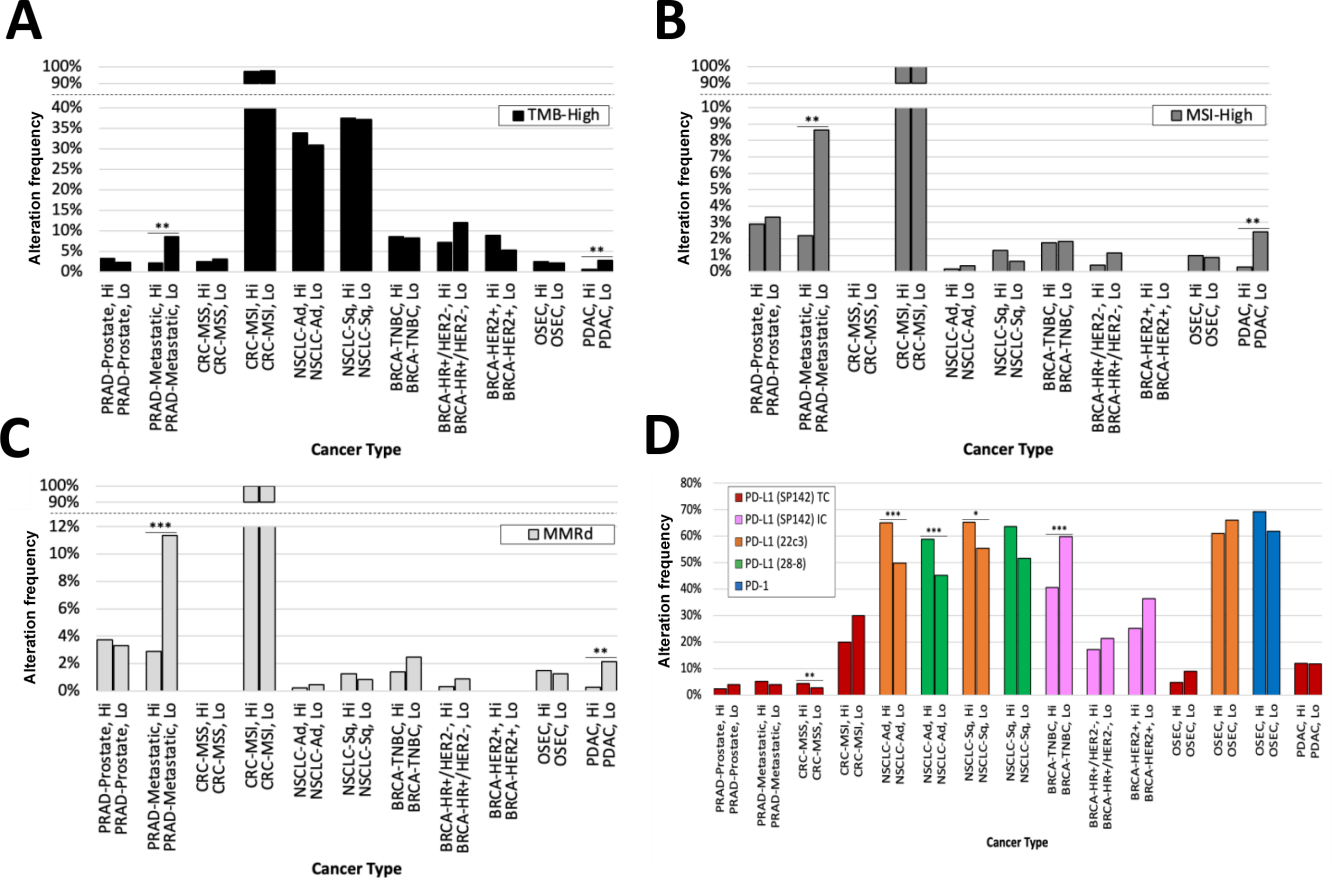
Independence of B7-H3 expression and immunotherapy biomarkers. Alteration frequency of TMB-high (**A**), MSI-high (**B**), MMRd (**C**), and PD-1/ PD-L1 status (**D**) across 11 different cancer types with high and low B7-H3 expression, including prostate cancer from a primary site (PRAD-Prostate), prostate cancer from a metastatic site (PRAD-Metastatic), colorectal cancer with microsatellite stability (CRC-MSS), colorectal cancer with microsatellite instability (CRC-MSI), non–small cell lung adenocarcinoma (NSCLC-Ad), non–small cell lung squamous carcinoma (NSCLC-Sq), triple-negative breast cancer (BRCA-TNBC), hormone receptor–positive/HER2-negative breast cancer (BRCA-HR+/HER2−), HER2-positive breast cancer (BRCA-HER2+), ovarian surface epithelial carcinoma (OSEC), and pancreatic carcinoma (PDAC). *, *q* < 0.05; **, *q* < 0.01; ***, *q* < 0.001. Cox proportional HRs were calculated for each comparison group with significance determined as *q* values of <0.05 using log-rank statistics.

Given that B7-H3 is within the same family of immune checkpoints as PD-L1 and CTLA-4, we sought to determine whether the well-established biomarkers for current ICIs are associated with B7-H3 status. We first focused on cancer types that demonstrated an association between B7-H3 expression and clinical outcome and then classified tumors in each cancer type into B7-H3-high versus –low based on the top and bottom quartiles in their respective cancer type. Examining genetic biomarkers for response to immunotherapy, we found that the level of B7-H3 is typically not associated with high TMB (TMB-high), MSI, or mismatch repair deficiency (MMRd; Fig. 3A–C). There are a few exceptions to this finding. First, in pancreatic adenocarcinomas, B7-H3–low tumors surprisingly display higher prevalence of TMB-high status (2.9% vs. 0.6%, *q* < 0.01), MSI-H status (2.4% vs. 0.3%, *q* < 0.01), and MMRd status (2.2% vs. 0.3%, *q* < 0.01), as well as in metastatic prostate cancers (samples taken from a metastatic site rather than the primary tumor) where B7-H3–low tumors displayed higher prevalence of MSI (8.7% vs. 2.2%, *q* < 0.01), TMB (8.7% vs. 2.1%, *q* < 0.01), and MMRd status (11.3% vs. 2.9%, *q* < 0.001). In examining B7-H3 expression in the context of PD-1/PD-L1 staining, we found that only lung cancers display positive correlations between B7-H3 and PD-L1 expression (*q* < 0.001), while triple-negative breast cancers display a negative correlation (*q* < 0.001; Fig. 3D). In addition, microsatellite stable (MSS) colorectal cancers showed a positive correlation between B7-H3 expression and PD-L1 expression.

### Altered Immune Contextures Underlie B7-H3–high versus low Tumors

**FIGURE 4:**
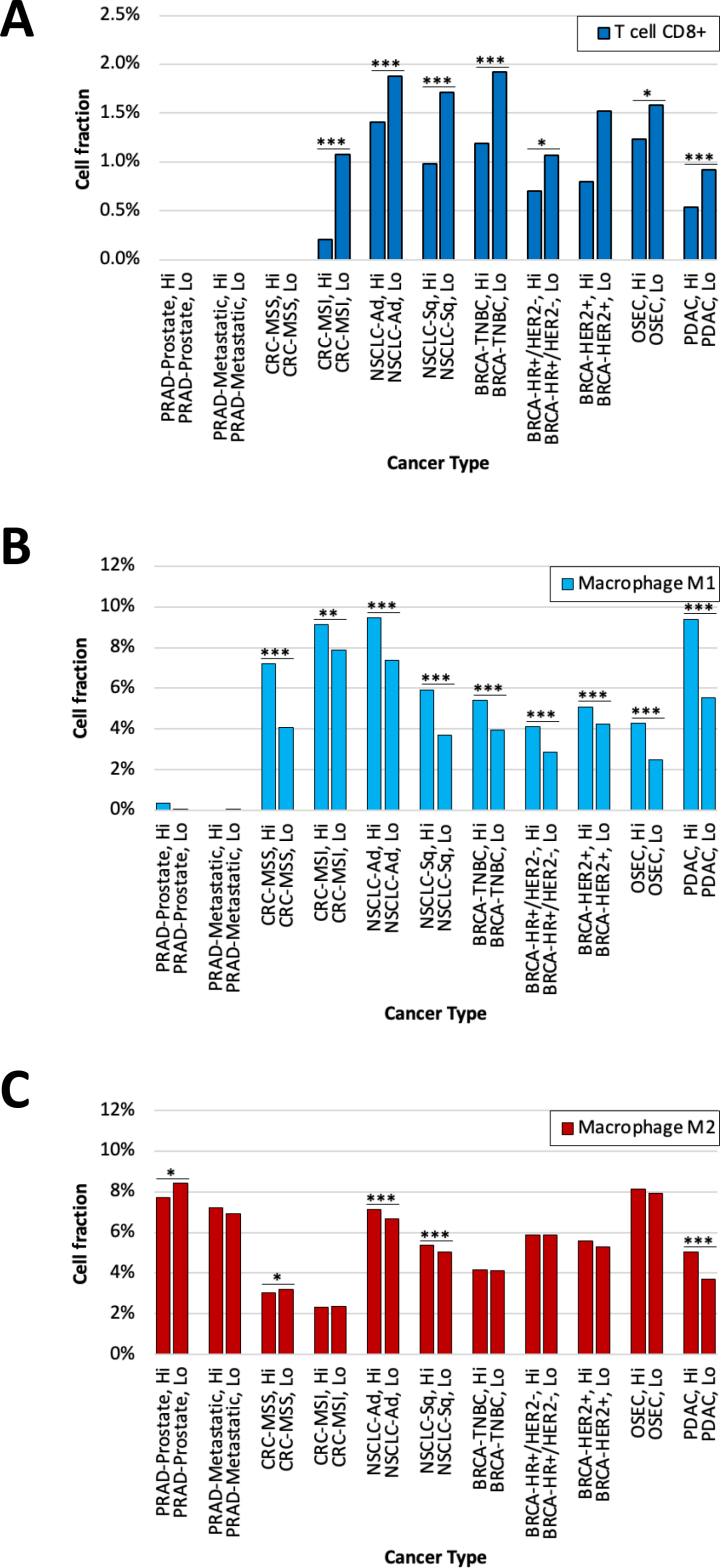
B7-H3 expression and immune cell landscape. Immune cell deconvolution using quanTIseq demonstrates differences in cell fraction of T cells CD8^+^ (**A**), macrophage M1 (**B**), and macrophage M2 (**C**) across 10 different cancer types with high and low B7-H3 expression. *, *q* < 0.05; **, *q* < 0.01; ***, *q* < 0.001.

Next, we sought to determine whether the level of B7-H3 expression was associated with unique tumor immune landscapes. Understanding these associations may inform the applicability of B7-H3 targeted immunotherapies that rely on activating antitumor immune responses. To deconvolute tumor-infiltrating immune cell fractions, we applied quanTIseq on bulk-RNA transcriptomic sequencing data (23). Indeed, we found that B7-H3–high tumors display divergent immune contextures relative to B7-H3–low tumors. In line with previous reports, we consistently observed B7-H3–high tumors to be depleted in CD8^+^ T cells, including in triple-negative breast cancers (1.2% vs. 1.9%, *q* < 0.0001), lung squamous cell carcinomas (1.0% vs. 1.7%, *q* < 0.0001), and lung adenocarcinomas (1.4% vs. 1.9%, *q* < 0.0001; Fig. 4A; refs. 29, 30). Interestingly, however, we found B7-H3–high tumors to be significantly and consistently enriched in proinflammatory M1 macrophages. We identified some of the greatest M1 densities in pancreatic adenocarcinomas (9.4% vs. 5.5%, *q* < 0.0001) and in both MSS and MSI colorectal cancers (7.2% vs. 4.1%, *q* < 0.0001 and 9.1% vs. 7.9%, *q* < 0.01, respectively; Fig. 4B; ref. 31). Conversely, we found immunosuppressive M2 macrophage populations to show minor differences between B7-H3–high versus –low tumors in select cancer types, including pancreatic adenocarcinomas (5.1% vs. 3.7%, *q* < 0.001), lung squamous cell carcinomas (5.4% vs. 5.1%, *q* < 0.001), and lung adenocarcinomas (7.1% vs. 6.7%, *q* < 0.001; Fig. 4C). These changes in M1 macrophage and CD8^+^ T-cell levels suggest that B7-H3 expression may influence immune-cell landscapes in a generally consistent manner across cancers.

### High B7-H3 Expression is Associated with More Aggressive Molecular Features

**FIGURE 5:**
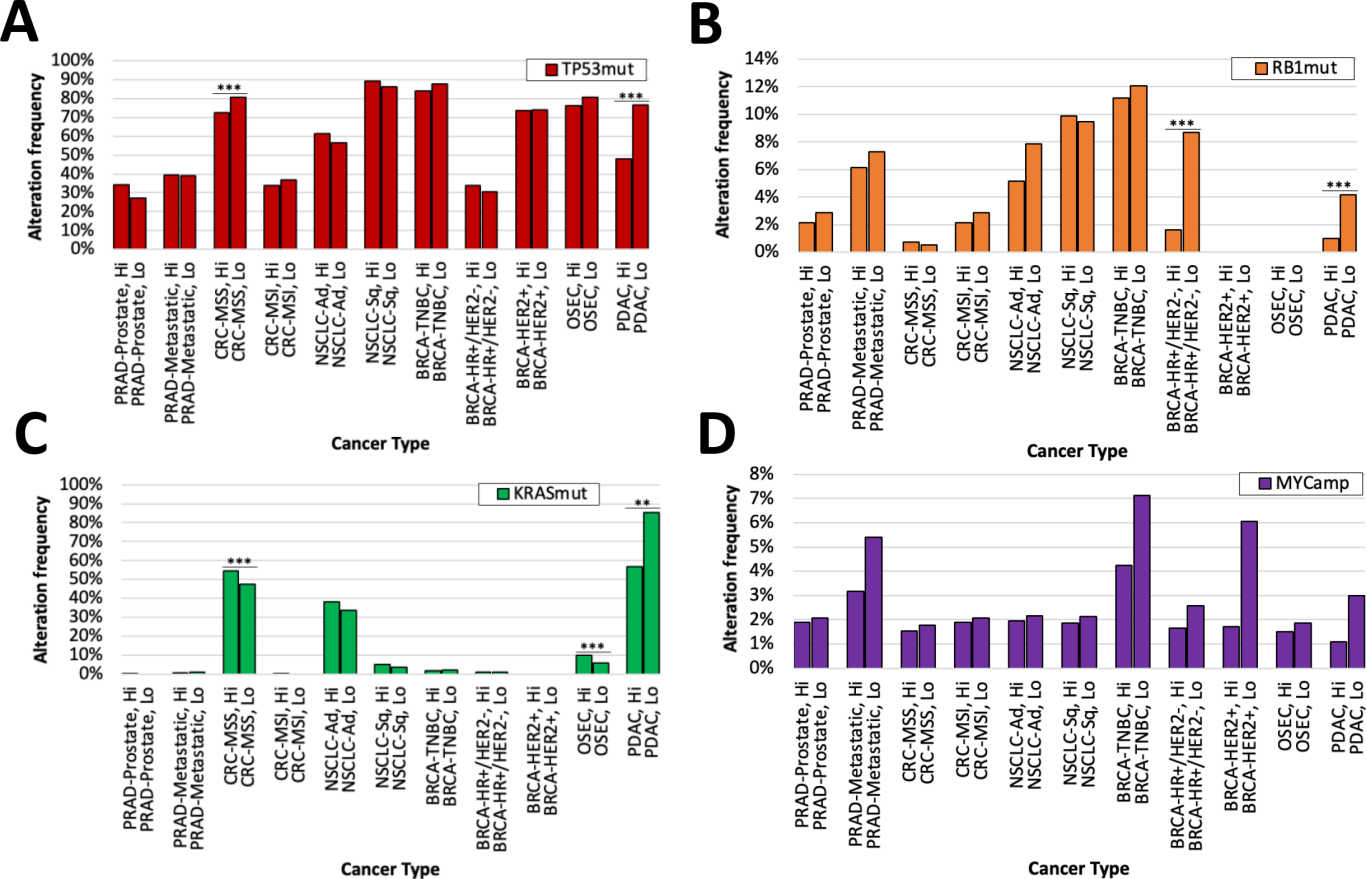
Association of B7-H3 expression with key driver mutations. Alteration frequency of *TP53* mutations (**A**), *RB1* mutations (**B**), *KRAS* mutations (**C**), *MYC* amplifications (**D**) of across 10 different cancer types with high and low B7-H3 expression. **, *q* < 0.01; ***, *q* < 0.001. Cox proportional HRs were calculated for each comparison group with significance determined as *q* values of <0.05 using log-rank statistics.

We next aimed to determine whether certain molecular features may be associated with elevated B7-H3 expression. Across cancer types, we found that B7-H3 expression is largely independent of pathogenic DNA alterations in known cancer genes (Fig. 5A–D). However, we did find associations between high versus low B7-H3 expression and the frequency of pathogenic alterations in key cancer genes *TP53*, *KRAS*, *RB1*, and *MYC*. Similar to our findings with immunotherapy biomarkers, we found these differences to be exemplified best in pancreatic adenocarcinomas, wherein B7-H3–high tumors displayed significant depletions for mutations in *KRAS* (56.8% vs. 85.5%, *q* < 0.0001), *TP53* (47.9% vs. 76.5%, *q* < 0.0001), and *RB1* (1.0% vs. 4.2%, *q* < 0.01). Of note, we also found B7-H3–high pancreatic adenocarcinomas to display fewer inactivating mutations in *RNF43*, which has recently been shown to accelerate the progression of *KRAS*-driven pancreatic neoplasia (32). We also found *RB1* mutations to be significantly depleted in B7-H3–high breast cancers that are hormone receptor (estrogen receptor/progesterone receptor) positive (HR+)/HER2 negative (1.6% vs. 8.7%, *q* < 0.0001). For MSS colorectal cancers, we found significant depletion of *TP53* mutations for B7-H3–high tumors (72.5% vs. 80.8%, *q* < 0.0001). In contrast, we observed *KRAS* mutations to be enriched in B7-H3–high ovarian serous carcinomas (9.8% vs. 6.0%, *q* < 0.05) as well as MSS colorectal cancers (54.4% vs. 47.4%, *q* < 0.0001).

**FIGURE 6:**
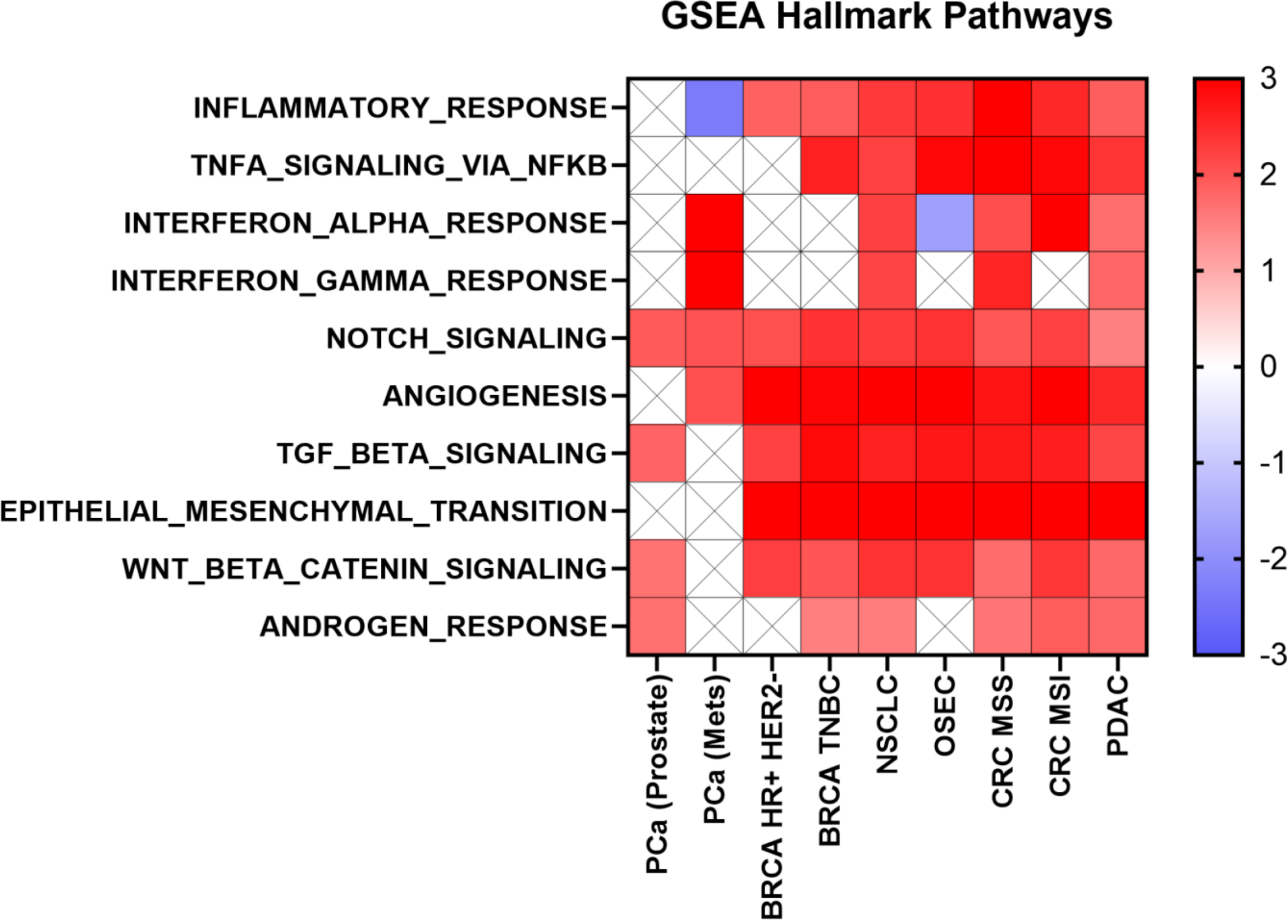
Association of B7-H3 expression and common hallmark mRNA signatures. GSEA with functional oncogenic pathways on several tumor types with high and low B7-H3 expression. Normalized enrichment scores are displayed. FDR < 0.05 based on values between −3 and 3. Boxes marked with “X” indicate that the enrichment of the pathway did not meet thresholds of significance in the tumor type.

To better understand whether high B7-H3 expression is indeed associated with more aggressive cancer phenotypes, we conducted gene set enrichment analyses (GSEA) to test associations of Hallmark gene sets with B7-H3–high versus –low tumors. Across cancer types, we found B7-H3–high tumors to be positively enriched for genes in the epithelial-to-mesenchymal transition (EMT) Hallmark gene set (*q* < 0.05; Fig. 6). In line with this, B7-H3–high tumors also displayed positive enrichment of Hallmark genes that are known to regulate EMT, including those reflecting WNT, NOTCH, and TGFβ signaling to be consistently upregulated in B7-H3–high tumors (*q* < 0.05; ref. 33). Interestingly, we also observed B7-H3–high tumors to be enriched for Hallmark angiogenesis signatures, concordant with previous reports of B7-H3 expression in pathologic angiogenesis (34–36).

Given the potential role of B7-H3 in immunoregulation, we explored associations with Hallmark gene sets reflecting immune signaling pathways. B7-H3–high tumors in most cancer types displayed positive enrichment of Hallmark inflammatory response gene sets, Hallmark IFNγ response gene sets, Hallmark TNFα response gene sets, and Hallmark IFNα response gene sets. Prior to GSEA, differential expression profiles were developed by comparing B7-H3–high and –low samples upon subsetting the transcriptomic data by the cancer types and subtypes we have focused on. Such altered signaling may be attributed to differences in immune contextures between B7-H3–high versus –low tumors. Nonetheless, we found divergent results in prostate cancers wherein B7-H3–high tumors displayed negative enrichment of Hallmark IFNγ, IFNα, and TNFα response gene sets. It is important to note, however, that in prostate cancers, we generally found few differences in immune contextures between B7-H3–high and –low tumors. Interestingly, comparing B7-H3–high versus –low specimens from metastatic prostate cancer relative to prostate biopsies from the primary site indicated distinct GSEA profiles. For instance, positive enrichment of Hallmark IFNγ, IFNα, and angiogenesis gene sets and negative enrichment of Hallmark inflammatory response gene sets were uniquely observed in B7-H3–high metastatic prostate cancers.

## Discussion

B7-H3 has become a promising target in many cancer types, with multiple targeted therapies currently in development. Here, we assess the clinicogenomic features associated with B7-H3 (*CD276*) expression to delineate which cancers may benefit the most from B7-H3 targeted therapies. We found a robust expression of B7-H3 in several major cancer types including prostate, colorectal, breast, pancreatic, ovarian, and lung cancers. While we find B7-H3 to be a prognostic marker for worse OS in most cancer types, we surprisingly find B7-H3 to be favorable in prostate and pancreatic adenocarcinoma. Although we do not identify consistent associations with mutational repertoires nor biomarkers of response to immunotherapies, we do find that tumors expressing high levels of B7-H3 have potential for myeloid-driven immune induction given enrichment in M1 macrophages and depletion of CD8^+^ T cells. These results indicate that immunomodulatory therapies targeting B7-H3 have potential to be effective against a variety of tumor types, including those that are not highly responsive to currently available treatments.

To our knowledge, this survey of B7-H3 expression represents the largest pan-cancer cohort to date. Although we find B7-H3 to be more robustly expressed in solid tumors than hematologic malignancies, it is still worthwhile to note that B7-H3 remains an attractive target currently being pursued in multiple myeloma and acute myeloid leukemia (37). Concordant with past reports, we demonstrate high B7-H3 expression to be associated with poorer prognosis in many cancer types, including NSCLCs and bone cancers. We also find divergent results wherein prostate adenocarcinomas display favorable outcomes with high B7-H3 expression. In addition, we must note that outside of prostate cancer, which included samples from metastatic sites, our analyses did not stratify tumor samples based on prior treatment status, metastatic versus primary disease, and biopsy site sequenced, which may impact tumor characteristics and clinical outcomes.

Importantly, we identify B7-H3 expression to be associated with altered immune-cell landscapes and dysregulated immune signaling pathways, highlighting its potential role in immune regulation. B7-H3 expression has previously been shown to limit CD8^+^ T-cell activation and proliferation, possibly explaining how B7-H3–high tumors in most cancer types are depleted in CD8^+^ T cells compared with B7-H3–low tumors (38, 39). Surprisingly, however, we detected an abundance of M1 macrophages in B7-H3–high tumors, positively shifting the ratio of M1/M2 macrophages in the tumor microenvironment. The M1/M2 macrophage ratio indicates switching of macrophage polarization from the M2 to M1 phenotype (40). B7-H3 has been suggested to promote M2 macrophage polarization, with the suppression of B7-H3 in ovarian cancer models leading to reduced numbers of infiltrating M2 macrophages (41, 42). Further studies are warranted to determine whether the enrichment of proinflammatory M1 macrophages in B7-H3–high tumors has any association with response in the context of immune-based therapies.

Despite the role of B7-H3 in immune regulation, our results suggest that B7-H3 expression is independent of other immunotherapy biomarkers such as TMB-high, MSI-high, MMRd status, as well as PD-L1 protein expression. As such, additional strategies to stratify potentially responsive patients are warranted as targeted therapies continue to be developed. We also identify few associations between B7-H3 expression and PD-1/PD-L1 in line with past reports of their poor coexpression (43). Nonetheless, this presents a potential opportunity in targeting solid tumors ineligible for PD-1/PD-L1–based therapies, as well as the possibility for dual targeting of B7-H3 and PD-1/PD-L1 which has previously shown antitumor activity in initial studies on head and neck cancers, NSCLC, and prostate cancer (44, 45).

Beyond B7-H3 in immune regulation, we also define its association with intrinsic tumor behaviors and other microenvironment characteristics. We find consistent evidence of B7-H3 being associated with upregulated EMT signatures, which may support the unfavorable prognoses associated with high B7-H3 expression in most cancer types. Prior *in vitro* studies demonstrated that modulation of B7-H3 in cervical, bladder, gastric, hepatocellular cancer cells promotes both migration and invasion (46–49). In addition, we observe B7-H3–high tumors to be consistently enriched for angiogenesis signatures, further supporting the role of B7-H3 in pathologic angiogenesis and in aiding tumor growth (36).

There are limitations to our study. First, we did not partition the tumors from each cancer type into subtypes, in which many would have distinct clinical and pathologic features and treatment strategies. Overall, we did not stratify all samples based on treatment, metastasis, or biopsy site which all have potential impacts on both clinical outcomes and tumor characteristics. There are some cancers which had very small patient numbers therefore limiting the conclusions that can be drawn for those specific tumor types. Our dataset comes from patients who are not enrolled in clinical trials, and we do not have individual patient data regarding tumor stage, grade, or treatment sequencing which is an inherent limitation of this study. Future work is warranted to uncover specific mechanisms and behavior of B7-H3 within such cancer types to partition tumors based on clinical features and treatment response that would be useful in future clinical trials. In addition, it is important to consider that high mRNA or even protein expression may not necessarily correlate with response to targeted therapy. For instance, high PD-L1 expression is not uniformly predictive of response to anti-PD-1/PD-L1 therapies in many cancer types (50). We also acknowledge that our OS analysis is unadjusted which may account for the favorable outcomes that are seen with high B7-H3 expression in some tumors such as in prostate cancer. In addition, our analysis of the immune cell landscape was based on bulk sequencing data using quanTIseq with tissues that were microdissected to maximize tumor tissue. These bulk sequencing methods have been benchmarked against single-cell approaches, and the quanTIseq algorithm does not include *CD276* as a gene signature, making it likely that B7-H3 expression seen originates from TCs rather than immune cells. Because there are currently no standardized B7-H3 IHC reagents for conducting staining of tumor tissue across cancer types, we utilized mRNA as a surrogate for protein expression based on the strong positive correlation between B7-H3 mRNA and protein levels that we found in our previous work (8). Nonetheless, future studies with interest in the detailed immune landscape should consider parallel approaches that may deconvolute the immune cell landscape with greater precision (23). It is important to note that this was a high-level descriptive analysis (association study) that falls short of delving deeply into tumor biology or immunology on a cancer-by-cancer basis, and thus serves only as a means to generate hypotheses that can then be tested in future mechanistic and clinical studies.

In conclusion, we report robust expression of B7-H3 in many common tumor types, including pancreatic, prostate, breast, ovarian, colorectal, and lung cancers. These tumors are often difficult to treat and therefore may benefit from B7-H3 targeted treatments. While we find that B7-H3–high tumors are not associated with conventional immunotherapy biomarkers, we observe consistent alterations in immune landscapes and signaling pathways. Furthermore, we demonstrate here that B7-H3 expression can reliably be extracted from clinical-grade transcriptomic data. Given that B7-H3 is highly expressed in many human malignancies, further studies should focus on expression and variability of B7-H3 within each cancer type in clinical scenarios, such as pretreatment and posttreatment and metastatic versus primary disease.

## Supplementary Material

Supplemental Figure 1 B7-H3 mRNA expression across cancers stratified by age (>/< 65 years old) and sex (male and female)
