# Supplementary material for: Pan-Cancer Interrogation of B7-H3 (CD276) as an Actionable Therapeutic Target Across Human Malignancies
Source: Cancer Res Commun. 2024 May 30;4(5):1369–79. doi: 10.1158/2767-9764.CRC-23-0546 (PMC11138391; doi:10.1158/2767-9764.CRC-23-0546)
Supplement: Supplemental Figure 1 — B7-H3 mRNA expression across cancers stratified by age (>/< 65 years old) and sex (male and female) [file crc-23-0546-s01.pptx]

## Slide 1
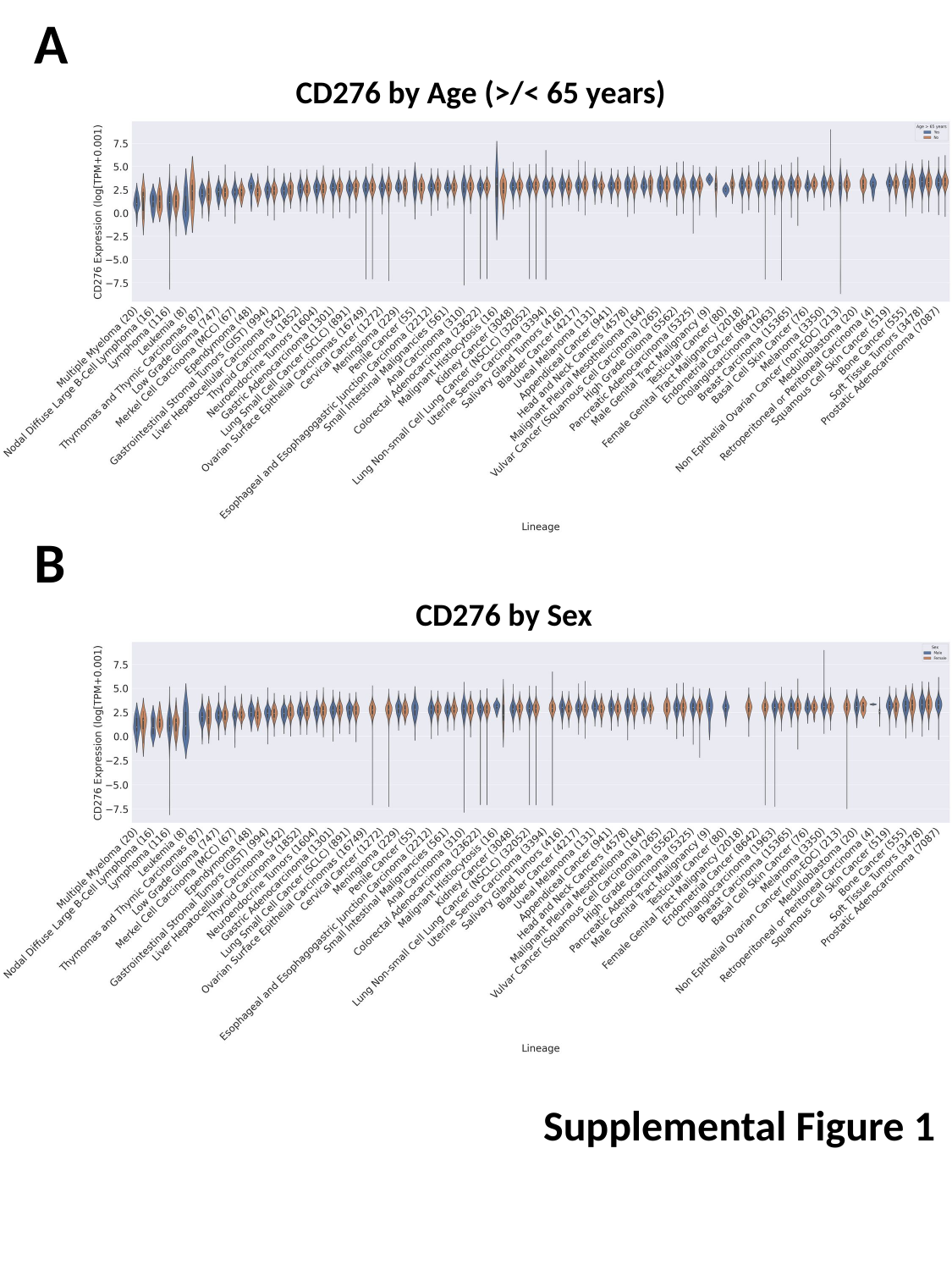

A
CD276 by Age (>/< 65 years)
B
CD276 by Sex
Supplemental Figure 1

## Slide 2
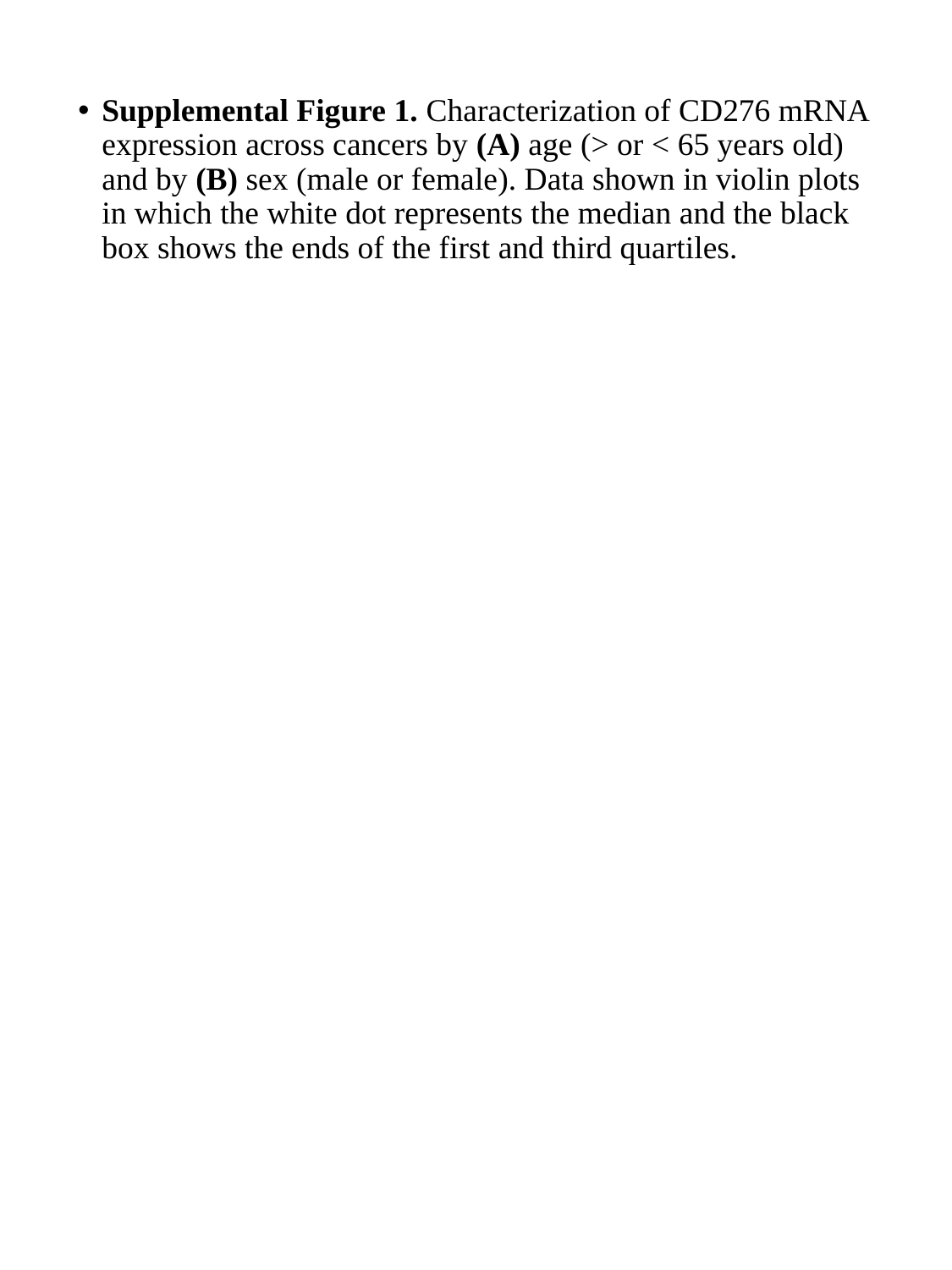

Supplemental Figure 1. Characterization of CD276 mRNA expression across cancers by (A) age (> or < 65 years old) and by (B) sex (male or female). Data shown in violin plots in which the white dot represents the median and the black box shows the ends of the first and third quartiles.
